# Lasting and Sex-Dependent Impact of Maternal Immune Activation on Molecular Pathways of the Amygdala

**DOI:** 10.3389/fnins.2020.00774

**Published:** 2020-08-11

**Authors:** Marissa R. Keever, Pan Zhang, Courtni R. Bolt, Adrienne M. Antonson, Haley E. Rymut, Megan P. Caputo, Alexandra K. Houser, Alvaro G. Hernandez, Bruce R. Southey, Laurie A. Rund, Rodney W. Johnson, Sandra L. Rodriguez-Zas

**Affiliations:** ^1^Department of Animal Sciences, University of Illinois at Urbana-Champaign, Urbana, IL, United States; ^2^Illinois Informatics Institute, University of Illinois at Urbana-Champaign, Urbana, IL, United States; ^3^High-throughput Sequencing and Genotyping Unit, Roy J. Carver Biotechnology Center, University of Illinois at Urbana-Champaign, Urbana, IL, United States; ^4^Neuroscience Program, University of Illinois at Urbana-Champaign, Urbana, IL, United States; ^5^Department of Statistics, University of Illinois at Urbana-Champaign, Urbana, IL, United States; ^6^Carl R. Woese Institute for Genomic Biology, University of Illinois at Urbana-Champaign, Urbana, IL, United States

**Keywords:** immune activation, pigs, RNA-seq, neuropeptides, glutamatergic pathway, GABAergic pathway

## Abstract

The prolonged and sex-dependent impact of maternal immune activation (MIA) during gestation on the molecular pathways of the amygdala, a brain region that influences social, emotional, and other behaviors, is only partially understood. To address this gap, we investigated the effects of viral-elicited MIA during gestation on the amygdala transcriptome of pigs, a species of high molecular and developmental homology to humans. Gene expression levels were measured using RNA-Seq on the amygdala for 3-week-old female and male offspring from MIA and control groups. Among the 403 genes that exhibited significant MIA effect, a prevalence of differentially expressed genes annotated to the neuroactive ligand–receptor pathway, glutamatergic functions, neuropeptide systems, and cilium morphogenesis were uncovered. Genes in these categories included corticotropin-releasing hormone receptor 2, glutamate metabotropic receptor 4, glycoprotein hormones, alpha polypeptide, parathyroid hormone 1 receptor, vasointestinal peptide receptor 2, neurotensin, proenkephalin, and gastrin-releasing peptide. These categories and genes have been associated with the MIA-related human neurodevelopmental disorders, including schizophrenia and autism spectrum disorders. Gene network reconstruction highlighted differential vulnerability to MIA effects between sexes. Our results advance the understanding necessary for the development of multifactorial therapies targeting immune modulation and neurochemical dysfunction that can ameliorate the effects of MIA on offspring behavior later in life.

## Introduction

The maternal immune response triggered by pathogens and other environmental stressors during gestation can also elicit an indirect response by the fetal immune cells (Kroismayr et al., 2004; Odorizzi and Feeney, 2016; Prins et al., 2018). Viral infection during gestation, for example, activates a cytokine-related signaling cascade, and molecules from this process can cross the placenta and reach the fetal brain. The resulting maternal immune activation (MIA) can impact fetal developmental processes and exert long-term postnatal effects in the offspring (Rutherford et al., 2014). The relationship between MIA and neurodevelopmental disorders, including schizophrenia spectrum disorders (SSD) and autism spectrum disorders (ASD), and neurodegenerative disorders, such as Alzheimer’s disease (AD), in offspring has been established (Knuesel et al., 2014; Canetta et al., 2016; Mattei et al., 2017). These diseases share some behavior symptoms, comorbidities such as eating disorders, and genetic and environmental (i.e., MIA) agents (Canitano and Pallagrosi, 2017). The previous neurological disorders have been associated with abnormal structure and dysregulation of the amygdala (Schumann et al., 2011; Fernandez-Irigoyen et al., 2014) and share genes and molecular mechanisms including histocompatibility complex (MHC) genes (Anders and Kinney, 2015), glutamatergic and GABAergic-associated genes (Bourgeron, 2009; Marin, 2012; Li et al., 2016), and mitochondrial activity processes (Pieczenik and Neustadt, 2007; Sragovich et al., 2017).

The fetal amygdala is susceptible to inflammatory signals, and the plasticity of this brain structure to MIA can lead to alterations of the developmental trajectory. These disruptions may have long-lasting and maladaptive consequences for the offspring, due to the significant role that the amygdala plays in many neurological pathways. Located in the forebrain, the amygdala influences social interaction, cognition, neuroendocrine, behavior, learning, memory, emotion, and autonomic systems. The amygdala also modulates the response of these processes to stressors, including pathogenic infections and those resulting from management practices, such as weaning (Tian et al., 2015). The amygdala experiences high uptake of gonadal hormones and is anatomically connected to other sexually dimorphic nuclei. Therefore, this brain region is involved in regulation of several dimorphic functions such as aggression, sexual behavior, gonadotropin secretion, and integration of olfactory information (Hines et al., 1992). Evidence supports the differential activation of the amygdala to stimuli between males and females (Killgore and Yurgelun-Todd, 2001), including differences in the sexual responses and emotional memory (Hamann, 2005), and differential vulnerability to insult (Baird et al., 2007). Due to the interconnected and multi-regulatory nature of this brain structure, insults to the amygdala can impact the individual’s social, locomotor, and feeding behavior (Petrovich and Gallagher, 2003); growth and reproductive physiology; health status; and immunological response to secondary stressors.

Recent studies lend support to the link between MIA and altered amygdala function (Carlezon et al., 2019). In mice, MIA elicited by polyinosinic:polycytidylic acid [Poly(I:C)] increased the synaptic strength of glutamatergic projections from the prefrontal cortex to the amygdala (Li et al., 2018). In open-field tests, mice exposed to MIA spent less time in the center and traveled a higher distance, indicative of a higher anxiety behavior incidence than the control counterparts. These findings suggest that the change in the balance between excitation (glutamatergic) and inhibition (feedforward GABAergic) modified the spike output of amygdala neurons, therefore affecting brain circuits that could regulate behavior in SSD and ASD. A candidate gene study of the effects of social stress during gestation reported that the expression of a corticotropin-releasing hormone receptor in the amygdala of 10-week-old pigs was higher in females than in males (Rutherford et al., 2014). This study concluded that prenatal stress substantially increased anxiety-related behaviors in female pigs. Studies of the impact of maternal stressors during gestation on specific amygdala molecular profiles and associated neurological or behavioral disorders in the offspring later in life highlight the complexity of the molecular mechanisms underlying the pathophysiology of MIA.

Research on the lasting effects of MIA in pigs complements the insights offered by rodent models (Antonson et al., 2019). The advantages of studying a pig model stem from the greater homology of humans to pigs, rather than to rodents, when considering organ physiology, size, development and, in particular, brain growth and development processes (Murphy et al., 2014). A pig model that has offered insights into MIA employs porcine reproductive and respiratory syndrome virus (PRRSV) to elicit MIA. This immune challenge activates the microglia (i.e., macrophage-like cells in the brain) and is associated with behavioral changes in neonatal pigs (Antonson et al., 2017, 2018).

The study of MIA elicited by PRRSV allows for the characterization of the impact of a live viral pathogen that self-replicates in the host, evoking extended activation of immune pathways. PRRSV challenge during gestation is a well-characterized, replicable, and effective method for inducing MIA in pigs (Antonson et al., 2017, 2018). In addition, PRRSV outbreaks impose a major economic burden to the livestock industry. PRRSV is an enveloped single-stranded RNA virus that infects alveolar macrophages, causing interstitial pneumonia and increased serum levels of the cytokines interleukin 1 beta, interleukin 6, and tumor necrosis factor alpha (Antonson et al., 2017). The persistent repercussions of MIA on the molecular pathways of the pig amygdala are yet to be investigated. Moreover, the potentially distinct vulnerability to the prolonged effects of MIA between sexes remains unknown.

The overarching goal of the present study is to advance the understanding of the impact of MIA on the molecular mechanisms of the amygdala. Three supporting objectives are explored: (a) characterization of prolonged transcriptome changes elicited by viral MIA in pigs, a species that has high neurodevelopmental homology with humans, and food production value; (b) identification of molecular pathways that present differential vulnerability to MIA between sexes; and (c) understanding the effect of MIA on molecular interactions assisted by gene network inference. The findings from these complementary analyses support the use of multiple therapeutic targets to ameliorate the potential detrimental effect of MIA on the offspring physiology and behavior.

## Materials and Methods

### Animal Experiments

All experimental procedures used published protocols (Antonson et al., 2017, 2018). The animal studies were approved by the Illinois Institutional Animal Care and Use Committee (IACUC) at the University of Illinois and are in compliance with the USDA Animal Welfare Act and the NIH Public Health Service Policy on the Humane Care and Use of Animals.

Camborough gilts born and raised at the University of Illinois at Urbana-Champaign herd were inseminated at 205 days of age using PIC 359 boar sperm (Antonson et al., 2017, 2018). All gilts were PRRSV negative and were moved at gestation day (GD) 69 into disease-containment chambers maintained at 22°C and a 12 h light/dark cycle with lights on at 7:00 AM. The gilts were fed daily 2.3 kg of a gestational diet and had *ad libitum* water access. One week after acclimation, four gilts were intranasally inoculated with live PRRSV strain P129-BV (School of Veterinary Medicine at Purdue University, West Lafayette, IN, United States) using 5 mL of 1 × 10^5^ median tissue culture infectious dose (TCID_50_) diluted in sterile Dulbecco’s modified Eagle medium (DMEM; 5 mL total volume). The four gilts in the Control group were intranasally inoculated with an equal volume of sterile DMEM. PRRSV inoculation corresponded to the last third of gestation in pigs and humans, during initiation of rapid fetal brain growth (Antonson et al., 2017, 2018). PRRSV and Control groups were housed in separate containment chambers.

The rectal temperatures and diet consumption of the gilts were recorded daily until farrowing (Antonson et al., 2017, 2018). The PRRSV-inoculated gilts were offered the maximum fed daily, and feed refusal was measured. The Control gilts were fed the same amount consumed by the PRRSV-inoculated gilts on the previous day. The daily body temperature and feed intake levels were compared using a mixed-effects model analyzed with PROC MIXED (SAS Institute Inc., Cary, NC, United States). The model included the effects of gilt treatment and replicate while accommodating for heterogeneity of variance between MIA groups.

Farrowing was induced with an intramuscular injection of 10 mg of Lutalyse (dinoprost tromethamine, Pfizer, New York, NY, United States) on GD 113 in consideration that the average gestation length is approximately 114 days (Antonson et al., 2017, 2018). Gilts farrowed in individual farrowing crates of standard dimensions (1.83 × 1.83 m). After farrowing, the gilts were fed twice a day up to 5 kg of a nutritionally complete diet for the lactating period and water remained available *ad libitum*. Pigs received intramuscular injections of iron dextran (100 mg/pig, Butler Schein Animal Health, Dublin, OH, United States) and Excede for Swine (25 mg/pig; Zoetis, Parsippany, NJ, United States) to control for respiratory diseases. The pigs remained with their mothers until PD 22. The body weight of pigs was measured daily and analyzed using the mixed-effects model in SAS, PROC MIXED (SAS Institute Inc., Cary, NC, United States). The model included the effect of MIA and the random effect of gilt, accommodating for heteroscedasticity between pig treatment and sex groups. The impact of MIA was studied at PD 22 because this is a common age to wean pigs. The study of transcriptome profiles from older pigs could be confounded with changes in diet and environment associated with weaning, while profiles from younger pigs would hinder the assessment of the prolonged effects of MIA.

### RNA Extraction and Sequencing

A balanced experimental design was studied, including 24 pigs evenly distributed between maternal PPRSV activated (MPA group of pigs) and Control gilts (CON group of pigs), each group encompassing males and females (denoted Ma and Fe, respectively). At PD 22, pigs were removed from the farrowing crate and anesthetized intramuscularly using a telazol:ketamine:xylazine drug cocktail (50 mg of tiletamine; 50 mg of zolazepam) reconstituted with 2.5 mL ketamine (100 g/L) and 2.5 mL xylazine (100 g/L; Fort Dodge Animal Health, Fort Dodge, IA, United States) at a dose of 0.03 mL/kg body weight, following protocols (Antonson et al., 2017).

Following anesthetization, pigs were euthanized using an intracardiac injection of sodium pentobarbital (86 mg/kg body weight, Fata Plus, Vortech Pharmaceuticals, Dearborn, MI, United States). Pig brains were extracted, the amygdalae were recognized using the stereotaxic atlas of the pig brain (Felix et al., 1999), dissected out, flash frozen on dry ice, and stored at −80°C following published protocols (Antonson et al., 2019). RNA was isolated using EZNA isolation kit following the manufacturer’s instructions (Omega Biotek, Norcross, GA, United States). The RNA integrity numbers of the samples were above 7.5, indicating low RNA degradation. The RNA-Seq libraries were prepared with TruSeq Stranded mRNAseq Sample Prep kit (Illumina Inc., San Diego, CA, United States). The libraries were quantitated by qPCR and sequenced on one lane on a NovaSeq 6000 for 151 cycles from each end of the fragments using NovaSeq S4 reagent kit. FASTQ files were generated and demultiplexed with the bcl2fastq v2.20 conversion software. Paired-end reads (150 nt long) were obtained, and the FASTQ files are available in the National Center for Biotechnology Information Gene Expression Omnibus (GEO) database (experiment accession number GSE149695).

### RNA Sequence Mapping and Differential Expression Analysis

The average Phred quality score of the reads assessed using FastQC (Andrews, 2010) was > 35 across all read positions, and therefore, no reads were trimmed. The paired-end reads from the individual samples were aligned to the *Sus scrofa* genome (version Sscrofa 11.1; Pruitt et al., 2007) using kallisto v0.43.0 (Bray et al., 2016) with default settings. The normalized (trimmed mean of *M*-values) gene expression values were described using a generalized linear model encompassing the effects of the MIA group (MPA or CON levels), sex (Fe or Ma levels), and MIA-by-sex interaction and analyzed using edgeR (version 3.14.0) in the R v. 3.3.1 environment (Robinson et al., 2010). Genes supported by > 5 transcripts per million (TPM) by each MIA–sex combination were analyzed to ensure adequate representation across comparisons.

Orthogonal pairwise contrasts between MIA and sex groups were evaluated in addition to testing for the effects of MIA-by-sex interaction and main effects of MIA and sex. The four groups compared in the contrasts, identified by treatment followed by the sex levels, are: MPA_Fe, MPA_Ma, CON_Fe, and CON_Ma. The *P*-values were adjusted for multiple testing using the Benjamini–Hochberg false discovery rate (FDR) approach (Benjamini and Hochberg, 1995).

### Functional Enrichment and Network Inference

Two complementary approaches were used to identify over-represented functional categories among the genes exhibiting differential expression across MIA and sex groups (Caetano-Anollés et al., 2015, 2016; Gonzalez-Pena et al., 2016a, b). Functional categories investigated included Gene Ontology (GO) biological processes (BPs), GO molecular functions (MF), and KEGG pathways. The Gene Set Enrichment Analysis (GSEA) approach implemented in the software package GSEA-P 2.0 (Subramanian et al., 2007) was used to identify category over-representation with gene over- and under-expressed while considering all genes analyzed. The normalized enrichment score (NES) of the categories in the Molecular Signature Database (MSigDB) was calculated using the maximum deviation of the cumulative sum based on the signed and standardized fold change. The statistical significance of the enrichment was assessed using the FDR-adjusted *P*-value computed from 1000 permutations.

The over-representation of functional categories was also evaluated among genes that exhibited a significant MIA-by-sex interaction or main effect using the Database for Annotation, Visualization and Integrated Discovery (DAVID 6.8) (Huang et al., 2009). The enrichment of Direct GO categories in the DAVID database was assessed. The *Sus scrofa* genome was used as the background for enrichment testing, and enrichment is reported using the Expression Analysis Systematic Explorer (EASE) score that was computed using a one-tailed jackknifed Fisher hypergeometric exact test. Functional categories were clustered based on gene annotation, and the statistical significance of clusters is summarized as the geometric mean of the -log_10_ EASE scores of the categories (Delfino et al., 2011; Serao et al., 2011; Delfino and Rodriguez-Zas, 2013).

### Weighted Gene Co-expression Network Analysis and Gene Network Visualization

An approach complementary to the identification of differentially expressed genes was used to uncover co-expression networks using Weighted Gene Co-expression Network Analysis (WGCNA) version 1.68 (Langfelder and Horvath, 2008). The input data were voom-transformed read count values generated using the limma package (version 3.40.2) (Ritchie et al., 2015) in R (version 3.6.1). Genes were filtered to remove those with low expression levels or no variation across samples per developer recommendations. The number of genes used for network analysis was 16,175 genes. Considering potential for interaction patterns, a sex-dependent soft-thresholding power was used to call for network topology analysis. The lowest power values that support a scale-free topology power used were 15 for the CON_Ma-MPA_Ma contrast and 27 for the MPA_Fe-MPA_Ma contrast. The Pearson correlation coefficient of the normalized expression values was used to identify modules of connected genes. The minimum module size was set to 30, with the deepSplit set to 2, and the mergeCutHeight set to 0.15. Module profiles were identified using the correlation between the eigengene of each module and pig group. Enrichment of functional categories among the genes in each module profile was explored with DAVID using the *Sus scrofa* genome as background, and testing included an FDR multiple test adjustment.

Further understanding of the impact of the MIA-by-sex interaction was gained through the reconstruction of gene networks using the BisoGenet package (Martin et al., 2010) in the Cytoscape platform (Shannon et al., 2003). Information from gene and protein interactions annotated in databases including BIOGRID, HPRD, DIP, BIND, INTACT, and MINT was used to visualize relationships between genes (Salwinski et al., 2004; Alfarano et al., 2005; Mishra et al., 2006; Stark et al., 2006; Kerrien et al., 2007; Licata et al., 2012). Networks highlighting differences in gene levels associated with MIA within sex (i.e., the contrasts MPA_Ma-CON_Ma and MPA_Fe-CON_Fe) were compared. The network framework includes genes that exhibited a significant MIA-by-sex interaction effect (FDR-adjusted *P* < 0.1) and are annotated to enriched functional categories. The framework genes were identified by full nodes with size reflecting the differential expression level between the MPA and CON groups. The network edges depict known molecular relationships curated in the BisoGenet databases. The framework genes were connected through correlated genes listed in the BisoGenet database of molecular interactions that did not reach significant MIA-by-sex interaction effect. The comparison of these networks offered insights into the simultaneous effect of MIA across interacting genes and enabled the detection of shared and distinct co-regulation patterns between MPA and CON pigs across sexes.

## Results

### Maternal Immune Activation and Sequencing Metrics

The differences between MPA and CON gilts in rectal temperatures and daily diet consumption indicated the activation of the maternal immune system in response to PRRSV. The difference in body temperature between CON and MPA gilts on GD 87 was -1.00°C (standard error 0.35°C; *P* < 0.005). The difference in feed refusal between CON and MPA gilts on GD 88 was -927.6 g (standard error 201.2 g; *P* < 0.0001). A significant increase in rectal temperatures and decrease in feed intake (*P* < 0.001) was observed within 48 h of inoculation and returned to baseline levels within 10 days for body temperature and within 14 days for feed intake. At 21 days of age, CON pigs were 1.20 kg heavier than MPA pigs (standard error = 0.5673; *P* < 0.089) while no significant sex or interaction effects were detected.

The sequencing of the 24 RNA samples produced 6.6 billion sequenced reads, and 69 million paired-end reads per sample. The number of reads was consistent across MIA and sex groups (coefficient of variation < 0.1), and the effects of MIA, sex, and MIA-by-sex interaction were tested on 16,175 genes that surpassed the minimum number of reads per MIA–sex combination.

### Transcriptome Changes Associated With Maternal Immune Activation That Are Sex-Dependent

Overall, 328 genes exhibited a significant (FDR-adjusted *P* < 0.1) MIA-by-sex interaction effect, and among these, 273 genes had a significant effect at FDR-adjusted *P* < 0.05. The profile of these genes indicated that the effect of MIA differed between females and males. Forty-six genes that presented a MIA-by-sex interaction effect are listed in Table 1 together with their expression pattern and *P*-value. The majority of the genes in Table 1, including neurotensin (NTS), displayed a reversal in the expression level between CON and MPA groups across sexes (i.e., opposite Log_2_[fold change] sign across sexes). An extended list including 328 genes that exhibited a MIA-by-sex interaction effect at FDR-adjusted *P* < 0.1 is provided in Supplementary File S1 Table A.

**TABLE 1:** Genes exhibiting significant (FDR-adjusted P-value < 0.1) maternal immune activation-by-sex interaction effect.

Gene symbol	*P*-value	^a^CON Fe-CON Ma	MPA Fe-MPA Ma	CON Fe-MPA Fe	CON Ma-MPA Ma	CON Fe-MPA Ma	CON Ma-MPA Fe
RGS16	<5E-11	–3.25	2.24	–2.44	3.06	–0.19	0.81
CGA	<5E-11	–5.86	0.36	0.27	6.49	0.63	6.13
POMC	<5E-11	–2.79	0.33	0.06	3.19	0.39	2.86
GPX3	<5E-11	–1.19	0.99	–0.37	1.81	0.63	0.82
RELN	<5E-11	–0.17	0.66	–0.74	0.09	–0.08	–0.57
VIPR2	<5E-11	–1.17	1.04	–1.03	1.18	0.00	0.14
ANKRD34C	<5E-11	–0.77	0.87	–0.71	0.93	0.16	0.06
GBP1	<5E-11	0.92	–0.32	–0.75	–1.99	–1.07	–1.66
GRM4	<5E-11	–0.97	0.91	–0.70	1.17	0.20	0.27
CCDC136	5.3E-09	–0.39	0.43	–0.15	0.67	0.29	0.24
SLC17A6	1.1E-08	–0.40	0.54	–0.16	0.77	0.37	0.23
BTBD11	4.4E-08	–0.62	0.45	–0.40	0.67	0.05	0.22
TTR	5.0E-08	–0.47	0.24	0.29	1.00	0.53	0.76
CACNA2D3	4.8E-07	–0.50	0.44	–0.26	0.68	0.18	0.23
CRHR2	2.8E-06	–0.24	1.09	–0.36	0.97	0.74	–0.12
NDNF	2.8E-06	–0.09	0.88	–0.70	0.27	0.18	–0.61
CXCL12	6.2E-06	–0.41	–0.01	–0.10	0.29	–0.11	0.30
USP43	6.4E-06	–0.64	0.43	–0.34	0.72	0.09	0.29
CCDC17	7.1E-06	0.74	–0.30	0.26	–0.40	0.03	0.12
KCNIP4	7.2E-06	–0.01	0.35	0.11	0.47	0.46	0.11
CAMK2N2	9.6E-06	–0.24	0.16	–0.23	0.18	–0.07	0.01
ALDH1A2	1.4E-05	–1.35	0.50	0.48	2.32	0.98	1.83
GRP	1.5E-05	–0.89	0.73	–0.61	1.02	0.12	0.28
PENK	1.6E-05	–0.10	0.46	–0.04	0.51	0.42	0.06
SYT12	2.9E-05	–0.16	0.32	–0.11	0.37	0.21	0.05
PTH1R	3.7E-05	–0.33	0.52	–0.51	0.34	0.01	–0.18
HBB	6.7E-05	–0.48	0.10	0.12	0.70	0.22	0.60
ESYT1	8.5E-05	–0.47	0.29	–0.27	0.49	0.02	0.20
EFHD1	9.6E-05	–0.31	0.42	–0.37	0.36	0.05	–0.06
BHLHE22	1.0E-04	0.06	–0.59	0.05	–0.61	–0.55	–0.01
ZFP37	1.2E-04	0.60	0.60	–0.19	–0.18	0.41	–0.78
SLC2A2	1.5E-04	0.08	0.50	–0.04	0.38	0.46	–0.12
THRSP	3.1E-04	–0.72	0.51	–1.24	–0.01	–0.73	–0.52
NR4A3	3.3E-04	–0.12	–0.01	–0.08	0.04	–0.08	0.04
LOC396781	4.4E-04	–0.75	1.36	–5.38	–3.27	–4.01	–4.63
C1QTNF1	4.5E-04	–0.37	0.32	–0.23	0.46	0.09	0.14
RAB27A	5.6E-04	–0.97	0.05	–0.25	0.77	–0.20	0.72
NTS	7.9E-04	–1.43	2.81	–1.78	2.47	1.03	–0.35
GVIN1	7.9E-04	0.80	–0.41	–1.01	–2.22	–1.42	–1.81
SSTR1	8.6E-04	0.11	–0.38	0.16	–0.33	–0.22	0.05
CCDC9B	8.8E-04	–0.24	0.35	–0.34	0.25	0.01	–0.10
CCDC33	1.4E-03	0.48	–0.39	0.49	–0.38	0.10	0.01
CCDC162P	1.4E-03	0.11	–0.48	0.36	–0.23	–0.12	0.25
PTH	1.4E-03	–0.03	0.29	–0.68	–0.36	–0.39	–0.65
SYNPO2L	1.5E-03	–0.28	0.59	–0.44	0.43	0.15	–0.16
CHGB	1.8E-03	–0.58	–0.16	0.37	0.79	0.21	0.96

*^*a*^Log_2_[fold change] between two maternal immune activation-sex groups: MPA, PRRSV-induced maternal immune activation; CON, control; Fe, females; Ma, males.*

Another frequent pattern among the genes that displayed a MIA-by-sex interaction effect was characterized by a consistent expression profile between CON and MPA across sexes, albeit the magnitude differed between sexes (Table 1). For example, glycoprotein hormones, alpha polypeptide (CGA) was over-expressed in CON relative to MPA, but the differential was higher in males than in females. Other genes presenting this pattern included guanylate-binding protein 1 (GBP1), transthyretin (TTR), aldehyde dehydrogenase 1 family member A2 (ALDH1A2), hemoglobin subunit beta (HBB), and basic helix-loop-helix family member e22 (BHLHE22).

Notable is the significant MIA-by-sex interaction effect on genes associated with neuropeptides and hormones, and genes that participate in glutamatergic processes. Genes under-expressed in MPA relative to CON males while presenting the opposite pattern in females (Table 1) included NTS, the neuropeptide gene proenkephalin (PENK), the neuropeptide gene gastrin-releasing peptide (GRP), the neuropeptide-related gene vasoactive intestinal peptide receptor 2 (VIPR2), corticotropin releasing hormone receptor 2 (CRHR2), neuron-derived neurotrophic factor (NDNF), reelin (RELN), glutamate metabotropic receptor 4 (GRM4), solute carrier family 17 member 6 (SLC17A6), calcium voltage-gated channel auxiliary subunit alpha 2 delta 3 (CACNA2D3), EF-hand domain family member D1 (EFHD1), glutathione peroxidase 3 (GPX3), parathyroid hormone 1 receptor (PTH1R), thyroid hormone responsive (THRSP), and CGA. The CGA gene codes for the alpha subunit protein of the hormones chorionic gonadotropin (CG), luteinizing hormone (LH), follicle-stimulating hormone (FSH), and thyroid-stimulating hormone (TSH).

### Functional and Network Analysis of Genes That Exhibit Sex-Dependent Associations With Maternal Immune Activation

The genes expressing significant MIA-by-sex interaction effects were analyzed for functional enrichment. Table 2 presents the clusters of most enriched and informative categories from the DAVID analysis, and the complete list of categories is in Supplementary File S1 Table B. The categories in Table 2 encompass genes presenting the most frequent interaction profile characterized by under-expression in CON females relative to males but over-expression in MPA females relative to males. These genes include KEGG Autoimmune thyroid disease (Cluster 1) and BP brain development (GO:0007420) (Cluster 4).

**TABLE 2:** Most enriched DAVID clusters and supporting functional categories (enrichment score ES > 1.3) among the genes presenting significant maternal immune activation-by-sex interaction effect.

^a^Category	Category identifier and name	*P*-value	^b^FDR *P*-value
**Cluster 1**	**ES = 2.74**		
KEGG	ssc05320:Autoimmune thyroid disease	2.90E-06	4.50E-04
BP	GO:000250∼Antigen processing and presentation of peptide or polysaccharide antigen via MHC class II	1.90E-03	3.40E-01
KEGG	ssc04514:Cell-adhesion molecules (CAMs)	2.30E-03	3.20E-02
KEGG	ssc05323:Rheumatoid arthritis	4.80E-03	5.60E-02
KEGG	ssc05164:Influenza A	2.00E-02	1.70E-01
**Cluster 2**	**ES = 1.9**		
BP	GO:0051050∼Positive regulation of transport	4.40E-03	3.50E-01
BP	GO:0051049∼Regulation of transport	4.50E-03	3.20E-01
BP	GO:0050801∼Ion homeostasis	1.30E-02	3.60E-01
BP	GO:0048878∼Chemical homeostasis	2.80E-02	4.80E-01
BP	GO:0030001∼Metal ion transport	4.50E-02	5.70E-01
**Cluster 3**	**ES = 1.75**		
BP	GO:0048871∼Multicellular organismal homeostasis	2.00E-04	3.70E-01
**Cluster 4**	**ES = 1.69**		
BP	GO:0061564∼Axon development	6.40E-05	1.70E-01
BP	GO:0007420∼Brain development	1.30E-03	3.10E-01

*^*a*^BP, biological process; KEGG, KEGG pathway. ^*b*^False discovery rate adjusted P-value.*

Enrichment results from GSEA complemented the findings from DAVID. Highly enriched informative categories among genes that have a MIA-by-sex interaction effect are presented in Table 3, and the extended list of categories is presented in Supplementary File S1 Table C. The categories in Table 3 support pathways in Table 2 including ion homeostasis (Table 2) and regulation of voltage-gated calcium channel activity processes (Table 3). Notably, the enrichment of the neuroactive ligand receptor interaction pathway and the hormone and neuropeptide activity processes include genes such as CGA and VIPR2 that were identified in Table 1.

**TABLE 3:** Enriched informative categories (NES > |1.3|) using GSEA among the genes based on the overall maternal immune activation-by-sex interaction.

^a^Category	Category identifier and name	^b^NES	*P*-value	^*c*^FDR *P*-value
KEGG	ssc04080:Neuroactive ligand receptor interaction	−1.84	<1.0E-10	8.3E-02
KEGG	ssc04912:GnRH signaling pathway	−1.83	<1.0E-10	9.9E-02
MF	GO:0005179∼Hormone activity	−1.80	<1.0E-10	2.2E-01
BP	GO:0006970∼Response to osmotic stress	−1.79	<1.0E-10	3.5E-01
BP	GO:0019221∼Cytokine mediated signaling pathway	−1.33	9.1E-02	5.4E-01
BP	GO:1901385∼Regulation of voltage gated calcium channel activity	−1.37	1.2E-01	5.4E-01
KEGG	ssc04020:Calcium signaling pathway	−1.34	1.2E-01	5.4E-01
BP	GO:0085029∼Extracellular matrix assembly	−1.31	1.8E-01	5.5E-01

*^*a*^MF, molecular function; KEGG, KEGG pathway; BP, biological process. ^*b*^Normalized enrichment score; negative values indicate genes under-expression in CON females relative to males but over-expression in MPA females relative to males. ^*c*^False discovery rate adjusted P-value.*

**FIGURE 1:**
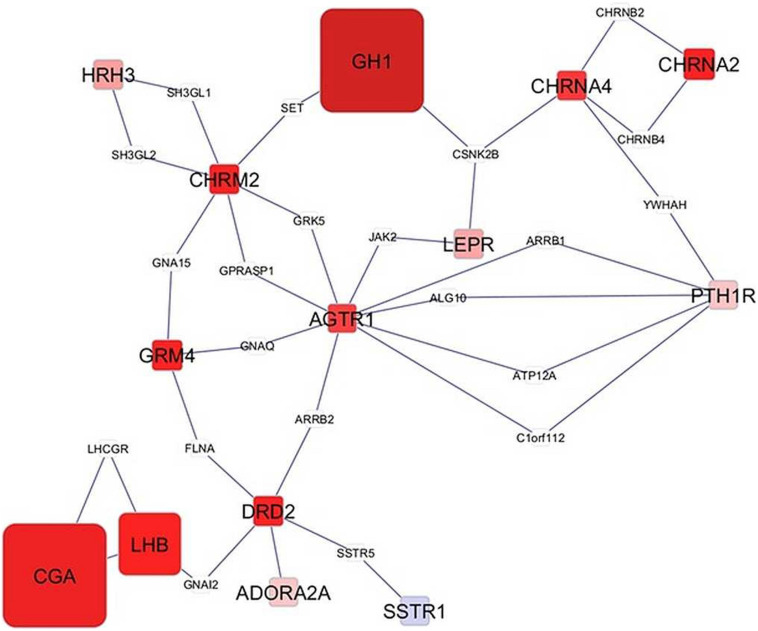
Network of genes differentially expressed in the amygdala of males (Ma) from control (CON) relative to maternal immune activation (MPA) (contrast between CON_Ma and MPA_Ma). Framework square node color: red and blue denote framework genes over- and under-expressed in CON relative to MPA pigs, respectively; framework node size: -Log_10_[*P*-value]; other genes connecting framework nodes were not differentially expressed in this study.

**FIGURE 2:**
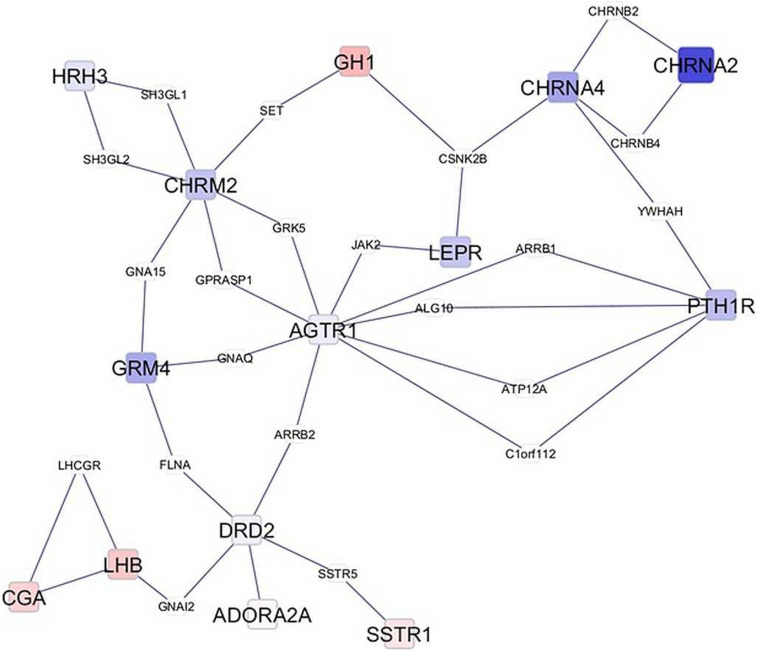
Network of genes differentially expressed in the amygdala of females (Fe) from control (CON) relative to maternal immune activation (MPA) (contrast between CON_Fe and MPA_Fe). Framework square node color: red and blue denote framework genes over- and under-expressed in CON relative to MPA pigs, respectively; framework node size: -Log_10_[*P*-value]; other genes connecting framework nodes were not differentially expressed in this study.

Network visualization furthered the understanding of the impact of MIA on the relationships among genes that exhibited a significant MIA-by-sex interaction effect. The networks in Figures 1, 2 depict the relationships between genes in the enriched neuroactive ligand receptor pathway that highlight the differential expression between CON and MPA in males and females (i.e., CON_Ma-MPA_Ma and CON_Fe-MPA_Fe contrasts), respectively. Red and blue rectangular nodes represent framework genes, and edges represent the known associations between genes based on curated databases of molecular interactions. Red and blue nodes denote over- or under-expression of the gene in CON relative to MPA, and the size is an inverse logarithmic function of the differential expression *P*-value. The simultaneous study of the differential expression pattern and connectivity among genes highlights the discrepancy in network modules elicited by MIA between the sexes.

### Transcriptome Changes Associated With Maternal Immune Activation

Overall, genes exhibited differential (FDR-adjusted *P* < 0.1) expression between MPA and CON pigs, irrespective of sex. Table 4 lists notable highly differentially expressed genes, and the complete list is in Supplementary File S1 Table D. The majority of these genes were over-expressed in MPA relative to CON pigs. Among the genes over-expressed in MPA compared to CON pigs were islet amyloid polypeptide (IAPP), ankyrin repeat domain 24 (ANKRD24), interferon-induced transmembrane protein 1 (IFITM1) and 3 (IFITM3), cathepsin C (CTSC), mitogen-activated protein kinase kinase 7 (MAP2K7), heparan sulfate-glucosamine 3-sulfotransferase 5 (HS3ST5), secreted phosphoprotein 1 (SPP1), immunoglobulin heavy chain (IGHG), and transforming acidic coiled-coil-containing protein 1 (TACC1). Among the genes under-expressed in MPA relative to CON pigs are insulin-like growth factor 2 (IGF2), cellular retinoic acid-binding protein 2 (CRABP2), and aldehyde dehydrogenase 1 family member A1 (ALDH1A1).

**TABLE 4:** Representative genes differentially expressed (FDR-adjusted *P*-value < 0.1) between pigs from control (CON) relative to PRRSV-treated (MPA) gilts.

Gene symbol	Gene name	^a^CON-MPA	*P*-value	^b^FDR *P*-value
IGHG	IgG heavy chain	–4.74	1.1E-31	<1.0E-10
IFITM3	Interferon induced transmembrane prot 3	–1.27	7.6E-11	6.2E-08
IGF2	Insulin-like growth factor 2	1.28	8.1E-07	2.7E-04
PAQR6	Progestin and adipoQ receptor family member 6	–1.02	1.9E-06	5.6E-04
RGS8	Regulator of G protein signaling 8	–0.88	8.6E-06	2.0E-03
NDNF	Neuron-derived neurotrophic factor	–1.27	1.5E-05	3.3E-03
HS3ST5	Heparan sulfate-glucosamine 3-sulfotransferase 5	–1.24	3.5E-05	6.8E-03
CTSC	Cathepsin C	–1.03	4.1E-05	7.7E-03
SPP1	Secreted phosphoprotein 1	–0.89	4.1E-05	7.7E-03
TACC1	Transforming acidic coiled-coil-containing protein 1	–1.55	5.5E-05	9.7E-03
IFITM1	Interferon induced transmembrane prot 1	–1.15	7.4E-05	1.2E-02
ALDH1A1	Aldehyde dehydrogenase 1 fam mem A1	0.79	9.7E-05	1.5E-02
PLEKHD1	Pleckstrin homology coiled-coil domain-containing D1	–0.99	5.6E-04	6.5E-02
HERC5	HECT and RLD domain-containing E3 ubiquitin ligase 5	–1.05	5.7E-04	6.6E-02
IAPP	Islet amyloid polypeptide	–1.36	7.0E-04	7.7E-02
ISLR	Immunoglobulin superfamily contain leucine rich repeat	1.13	8.3E-04	8.5E-02
ANKRD24	Ankyrin repeat domain 24	–0.73	8.7E-04	8.9E-02
MAP2K7	Mitogen-activated protein kinase kinase 7	–0.64	9.9E-04	1.0E-01

*^*a*^Log_2_[fold change] between CON and MPA pigs. ^*b*^False discovery rate adjusted P-value.*

### Functional Analysis of Genes Associated With Maternal Immune Activation

Table 5 presents the top significant clusters of informative enriched categories from the DAVID analysis of genes differentially expressed between MPA and CON groups across sexes (the extended list of categories is presented in Supplementary File S1 Table E). Some categories identified by the DAVID analysis are consistent with the categories detected at more significant levels among the genes presenting an MIA-by-sex interaction effect (Table 2) and include the BP angiogenesis (GO:0001525) and KEGG autoimmune thyroid disease and Epstein–Barr virus infection pathways (Table 5). Also enriched (Supplementary File S1 Table E) were the BP homeostatic (GO:0042592), MF ion binding (GO:0043167), and BP anatomical structure formation in morphogenesis (GO:0048646).

**TABLE 5:** Clusters of enriched functional categories (enrichment score ES > 1.3) among the genes presenting significant maternal immune activation effect, and representative categories identified using DAVID.

^a^Category	Category identifier and name	*P*-value	^b^FDR *P*-value

Cluster 1	ES = 1.51		
BP	GO:0048646∼anatomic structure formation in morphogenesis	3.7E-03	9.7E-01
BP	GO:0001525∼angiogenesis	8.4E-02	9.8E-01

**Cluster 2**	**ES = 1.40**		

KEGG	ssc05330:Allograft rejection	1.7E-02	8.0E-01
KEGG	ssc05169:Epstein–Barr virus infection	2.3E-02	6.7E-01
KEGG	ssc05320:Autoimmune thyroid disease	3.0E-02	6.1E-01

*^*a*^BP, biological process; KEGG, KEGG pathway. ^*b*^False discovery rate adjusted P-value.*

The GSEA enrichment results within the gene expression patterns of CON relative to MPA groups complemented the findings from DAVID. The most informative enriched categories are presented in Table 6, and the extended list of categories is presented in Supplementary File S1 Table F. Enriched clusters of genes over-expressed in CON relative to MPA detected by GSEA were the BP enrichment of microtubule bundle formation (GO:0001578) and cilium morphogenesis (GO:0060271).

**TABLE 6:** Enriched informative categories (NES > |1.3|) among the genes differentially expressed between pigs from the control relative to the maternal immune activation group using GSEA.

^a^Category	Category identifier and name	^b^NES	*P*-value	^*c*^FDR *P*-value
BP	GO:0001578∼Microtubule bundle formation	2.03	<1.0E-10	<1.0E-08
BP	GO:0060271∼Cilium morphogenesis	2.01	<1.0E-10	<1.0E-08
BP	GO:0044782∼Cilium organization	1.97	<1.0E-10	<1.0E-08
BP	GO:0035082∼Axoneme assembly	1.94	<1.0E-10	1.8E-04
BP	GO:0003341∼Cilium movement	1.94	<1.0E-10	1.4E-04

*^*a*^BP, biological process. ^*b*^Normalized enrichment score, positive values refer to genes under-expressed in maternal immune activated relative to control pigs. ^*c*^False discovery rate adjusted P-value.*

### Transcriptome Differences Between Sexes Independent of Maternal Immune Activation

Overall, 150 genes were differentially expressed between males and females (FDR-adjusted *P* < 0.05). These genes exhibited a consistent differential expression between sexes, irrespective of the MIA group. The complete list of genes differentially expressed between sexes at FDR-adjusted *P* < 0.1 is available in Supplementary File S1 Table G, and the majority were over-expressed in males relative to females. Among the previous genes, excluding those that presented MIA-by-sex interaction effect, a selection of informative genes is listed in Table 7. Genes over-expressed in males relative to females included eukaryotic translation initiation factor 1A, Y-linked (EIF1AY), leptin receptor (LEPR), luteinizing hormone beta polypeptide (LHB), LIM homeobox 9 (LHX9), luteinizing hormone beta polypeptide (LHB), and immunoglobulin family member 1 (IGSF1).

**TABLE 7:** Informative genes presenting significant differential expression between males (Ma) and females (Fe).

Gene symbol	Gene name	^a^Ma-Fe	*P*-value	^b^FDR *P*-value
EIF1AY	Eukaryotic translation initiation factor 1A, Y-linked	13.68	<1.0E-08	<1.0E-08
LHX9	LIM homeobox 9	3.38	<1.0E-08	<1.0E-08
LHB	Luteinizing hormone beta polypeptide	3.92	<1.0E-08	<1.0E-08
TRPC3	Transient receptor potential cation channel C3	1.83	1.7E-07	4.4E-05
WNT3	Wnt family member 3	1.72	9.0E-07	2.0E-04
IGSF1	Immunoglobulin superfamily member 1	0.85	6.4E-06	1.3E-03
NPM2	Nucleophosmin/nucleoplasmin 2	1.79	1.8E-05	2.8E-03
RORA	RAR-related orphan receptor A	0.74	2.0E-04	2.4E-02
LEPR	Leptin receptor	0.62	8.8E-04	8.4E-02

*^*a*^Log_2_[fold change] between male and female offspring. ^*b*^False discovery rate adjusted P-value.*

Informative categories among the DAVID clusters of enriched categories for the genes differentially expressed between sexes are listed in Table 8 (a complete list is available in Supplementary File S1 Table H). The previous categories include BP gland development (GO:0048732), response to hormone (GO:0009725), and brain development (GO:0007420).

**TABLE 8:** Clusters of informative enriched functional categories (enrichment score ES > 1.3) among the genes differentially expressed between sexes identified using DAVID.

^a^Category	Category identifier and name	*P*-value	^b^FDR *P*-value
**Cluster 1**	**ES = 1.82**		
MF	GO:0005201∼extracellular matrix structural constituent	3.0E-02	7.9E-01
**Cluster 2**	**ES = 1.74**		
KEGG	ssc04080:Neuroactive ligand-receptor interaction	2.7E-06	2.4E-04
BP	GO:0009725∼response to hormone	7.3E-04	9.4E-01
**Cluster 3**	**ES = 1.74**		
BP	GO:0048732∼gland development	1.7E-03	5.7E-01
**Cluster 4**	**ES = 1.45**		
BP	GO:0007420∼brain development	1.1E-01	7.9E-01
BP	GO:0030900∼forebrain development	1.9E-01	8.7E-01
**Cluster 5**	**ES = 1.41**		
MF	GO:0020037∼heme binding	6.2E-02	9.1E-01
MF	GO:0005506∼iron ion binding	9.3E-02	9.3E-01
**Cluster 6**	**ES = 1.35**		
BP	GO:0009790∼embryo development	5.8E-03	5.4E-01
BP	GO:0090596∼sensory organ morphogenesis	8.9E-03	5.1E-01

*^*a*^BP, biological process; MF, molecular function; KEGG, KEGG pathway. ^*b*^False discovery rate adjusted P-value.*

### Weighted Gene Co-expression Network Analysis

The WGCNA study of the correlation between expression and experimental factors was based on the log-transformed TPM expression level of 16,175 genes. This study identified 62 modules of expression correlated with MIA groups among males. The number of genes in each module ranges from 32 to 1381; Supplementary File S1 Figure A depicts the relationship between gene modules using a dendrogram. The correlation (corr) between the eigengene expression profile and maternal treatment in males is depicted in Supplementary File S1 Figure B. A fairly even distribution of positive and negative correlation estimates was observed. The genes in the modules pale violet red (corr = –0.58, *P* < 0.03) and gray60 (corr = –0.59, *P* < 0.06) presented a strong negative correlation, indicating that low expression levels were associated with MPA.

The enrichment analysis of the genes in the WGCNA modules provided additional insights into the processes impacted by MIA. The extended list of enriched categories across modules is available in Supplementary File S1 Table I. Table 9 lists representative clusters of enriched categories in the gene modules that were highly correlated with MIA differences in males. Complementary categories that were enriched in modules encompassing gene patterns negatively correlated with MPA include the KEGG AD and oxidative phosphorylation pathways in the pale violet red3 and the gray60 modules, MF NADH dehydrogenase activity (GO:0003954) in the gray60 module, and the KEGG ribosomal pathway and associated GO processes in the light yellow module. Confirming the enrichment results from the previous differential expression analysis (Table 2 and Supplementary File S1 Table B), categories enriched in modules encompassing gene patterns negatively correlated with MPA include ion transport (also in Table 2 and Supplementary File S1 Table H) in the pale violet red3 and gray60 modules, and oxidoreductase activity in the gray60 module (also in Supplementary File S1 Table H).

**TABLE 9:** Clusters of enriched functional categories (enrichment score ES > 1.3) among the genes in modules presenting a significant correlation with maternal immune activation (MPA) relative to control within males using DAVID.

^a^Category	Category identifier and name	*P*-value	^b^FDR *P*-value

MODULE	Gray60 (low expression in MPA)		
**Cluster 1**	**ES = 14.5**		
KEGG	Ssc05010:Alzheimer’s disease	1.3E-18	9.1E-17
KEGG	Ssc05012:Parkinson’s disease	9.8E-18	4.7E-16
**Cluster 2**	**ES = 7.32**		
MF	GO:0016651∼oxidoreductase activity, acting on NAD(P)H	3.6E-10	1.5E-07
MF	GO:0003954∼NADH dehydrogenase activity	3.4E-09	6.8E-07
**Cluster 3**	**ES = 6.41**		
BP	GO:0042775∼mitochondrial ATP synthesis electron	4.0E-10	8.6E-07
BP	GO:0046034∼ATP metabolic process	8.0E-10	8.7E-07
BP	GO:0009116∼nucleoside metabolic process	7.8E-08	9.4E-06

**MODULE**	**Light yellow (low expression in MPA)**		

**Cluster 1**	**ES = 4.18**		
KEGG	ssc03010:Ribosome	3.7E-06	5.9E-05
BP	GO:0006412∼translation	9.5E-06	1.7E-02
**Cluster 1**	**ES = 2.49**		
BP	GO:0007006∼mitochondrial membrane organization	5.1E-04	7.8E-02
BP	GO:0007007∼inner mitochondrial organization	1.1E-02	3.5E-01

*^*a*^BP, biological process; MF, molecular function; KEGG, KEGG pathway. ^*b*^False discovery rate adjusted P-value.*

The WGCNA study identified 61 modules of expression correlated with sex among MPA pigs. The number of genes in each module range from 34 to 2082 and Supplementary File S1 Figure C depicts the dendrogram of modules. Positive correlation estimates that indicate higher expression in male than female were identified in the ivory (corr = 0.65, *P* < 0.03) and antique maroon (corr = 0.62, *P* < 0.04) modules, whereas negative correlations denoting lower expression in male were identified in sienna3 (corr = –0.65, *P* < 0.03) (Supplementary File S1 Figure D). Table 10 lists representative clusters of enriched functional categories in these modules. The extended list of categories and modules is in Supplementary File S1 Table J. Within the ivory module of positively correlated gene patterns, the BP synapse (GO:0007416) and neural development (GO:0007399) categories were enriched.

**TABLE 10:** Clusters of enriched functional categories (enrichment score ES > 1.3) among the genes in modules presenting a significant correlation with sex within the maternal immune activation treatment using DAVID.

^a^Category	Category identifier and name	*P*-value	^b^FDR *P*-value

MODULE	Sienna3 (low expression in males)		
**Cluster 1**	**ES = 1.58**		
BP	GO:0044255∼cellular lipid metabolic process	1.1E-02	1.0E+00
BP	GO:0044283∼small molecule biosynthetic process	3.3E-02	9.8E-01

**MODULE**	**Ivory (high expression in males)**		

**Cluster 1**	**ES = 1.47**		
BP	GO:0007416∼synapse assembly	8.1E-03	1.0E+00
BP	GO:0007399∼nervous system development	2.7E-02	9.1E-01

*^*a*^BP, biological process; MF, molecular function; KEGG, KEGG pathway. ^*b*^False discovery rate adjusted P-value.*

## Discussion

The present study uses a validated model of MIA triggered by a live viral (i.e., PRRSV) infection during a critical neurodevelopmental stage (Antonson et al., 2017) to gain innovative insights into sex-dependent molecular changes in the amygdala. The changes in gilt body weight and temperature within 2 weeks postinfection were consistent with the mode of action of the PRRSV and previous reports (Antonson et al., 2017). These results indicate that the PRRSV-infected gilts experienced extended activation of inflammatory pathways during gestation. The present study characterized the prolonged effect of the MIA, 60 days after exposure, on 3-week-old offspring.

Our study identified patterns of differential gene expression and prevalently dysregulated gene networks and processes, some of which have been reported in clinical and preclinical studies of AD, ASD, and SSD (Malkova et al., 2012; Xuan and Hampson, 2014; Aavani et al., 2015). These disorders have been associated with MIA and amygdala functions, yet the corresponding neurological and molecular changes have been studied mostly using pathogen mimetic challenges on rodents (Xuan and Hampson, 2014; Aavani et al., 2015). Moreover, our study identified sex-dependent molecular patterns that are consistent with the differential prevalence and symptoms of ASD, SSD, and MIA-related disorders between sexes reported in rodent and human studies (Wischhof et al., 2015). For example ASD tends to be more prevalent in young males (Kirsten et al., 2010; Haida et al., 2019), while SSD tends to be more prevalent in females (Bale et al., 2010; Bale, 2011). Similarly, lower sociability and preference for social novelty were observed in 2 weeks old pigs from gilts inoculated with PRRSV at GD 76 than from control gilts (Antonson et al., 2017). A discussion of the molecular mechanisms impacted by MIA can offer insights into therapies to ameliorate the lasting effects on physiology and behavior.

### Sex-Dependent Transcriptome Changes Associated With Maternal Immune Activation

The evaluation of the 328 genes presenting a significant MIA-by-sex interaction effect augmented the understanding of the differential response of transcripts to MIA between sexes (Table 1 and Supplementary File S1 Table A). The majority of the previous genes were under-expressed in MPA relative to CON males, and the profile in females was opposite or less extreme. Many genes presenting a significant MIA-by-sex effect code for neuropeptides and hormones, or participate in glutamatergic processes.

The lower NTS levels in the amygdala of male rats associated with lower conditioned place preference (Laszlo et al., 2010) is consistent with the lower level of NTS transcripts in MPA males observed in the present study (Table 1). The under-expression of neuropeptide gene POMC in MPA relative to CON males (Table 1) may be associated with the changes in POMC-related peptide transmission that has been reported in the brains of patients diagnosed with SSD (Jamali and Tramu, 1997). The under-expression of the neuropeptide gene PENK in MPA relative to CON males (Table 1) is in agreement with lower levels of PENK expression in the brains of mice models of SSD (Gottschalk et al., 2013). PENK was also differentially expressed in the amygdala of an ASD rat model using prenatal valproic acid exposure (Oguchi-Katayama et al., 2013). Valproic acid treatment during pregnancy was associated with a sevenfold increase in ASD incidence, social difficulties, and reduced attention (Roullet et al., 2013). Also, CACNA2D3 was under-expressed in the amygdala of rats exposed prenatally to valproic acid and coincided with social behavior abnormalities including heightened anxiety (Barrett et al., 2017). In the present study, the interaction pattern of CACNA2D3 was characterized by under-expression in MPA relative to CON males (Table 1).

The over-expression of the neuropeptide receptor VIPR2 in the amygdala of MPA relative to CON females (Table 1) is consistent with reports that duplications in this gene confer a significant risk for SSD (Morris and Pratt, 2014). The differential expression of the VIP receptor is particularly important because GABAergic interneurons in the amygdala express the neuropeptide VIP that facilitates cell firing (Rhomberg et al., 2018) and maintains the balance of pro- and anti-inflammatory cytokines (Martinez et al., 2020). The under-expression of GPX3 in MPA relative to CON males (Table 1) is consistent with the association between GPX3 gene expression and SSD (Zhao et al., 2018).

The sex-dependent response to MIA of hormone receptor CRHR genes detected in our study (Table 1) has been also reported by others. The expression of CRHR1 in the amygdala of 10 weeks old female pigs from sows exposed to a social stressor during mid-gestation was higher than in pigs from control gilts, whereas no stressor effects were observed in males nor in the expression of CRHR2 (Rutherford et al., 2014). Also, the expression of CRHR was associated with SSD (Mistry et al., 2013). The alignment between the profiles of the CRHR2 and neuropeptide GRP genes observed in our study (Table 1) is in agreement with reports of simultaneous release of CRH and GRP in the amygdala of rats elicited by the stress hormone corticosterone (Merali et al., 2008). The over-expression of the parathyroid hormone receptor PTH1R gene in response to MIA (Table 1) is consistent with the over-expression of this gene in SSD and AD (Ibanez et al., 2014). The over-expression of the functionally related thyroid hormone responsive gene THRSP in MPA relative to CON females further supports the PTH1R pattern (Munshi and Rosenkranz, 2018).

Several genes in the glutamatergic and GABAergic pathways displayed sex-dependent MIA effects (Table 1). This shared pattern may stem from pro-inflammatory cytokines intensifying glutamatergic release by the amygdala in a sex-dependent manner. The glutamate receptor GRM4 was under-expressed in MPA relative to CON males, while a less extreme and opposite trend was detected in females (Supplementary File S1 Table A). Supporting our finding, the expression of glutamate receptor genes was lower in SSD brains (Meador-Woodruff and Healy, 2000). The pattern differences between sexes could be connected with differences in gene expression across sexes in multiple GRM genes including GRM4 in association with behavior disorders (Gray et al., 2015). The expression pattern of SLC17A6, a gene in the glutamatergic pathway, was consistent with that of GRM4 (Table 1). Moreover, NDNF and RELN (two genes in the GABAergic pathway) displayed similar expression patterns in our study in agreement with previous reports (Hou and Capogna, 2018). NDNF interneurons evoke inhibitory postsynaptic potentials mediated by GABA receptors. Also, the GABAergic gene RELN has been associated with ASD and SSD (Canitano and Pallagrosi, 2017) and is under-expressed in the prefrontal cortex of subjects diagnosed with SSD (Fatemi et al., 2005). Consistent with our results, genes that regulate neural migration of GABAergic interneurons were under-expressed in the brain of offspring from rats exposed to lipopolysaccharide (LPS)-induced MIA (Oskvig et al., 2012). The activity of GABA and glutamate on serotonergic neurons is modulated by chemokine ligands such as CXCL12 (Stuart and Baune, 2014), and consistent with this interaction, CXCL12 and CXCL13 were under-expressed in MPA relative to CON males (Table 1 and Supplementary File S1 Table A). Our results are also consistent with findings that LPS injection of adult mice dysregulated CXCL12, which in turn increased glutamatergic release in the amygdala, and anxiety-like behavior occurrence (Yang et al., 2016). EFHD1, a calcium binding protein associated with synaptic transmission and levels of gamma glutamyltransferase was over-expressed in MPA relative to CON females (Supplementary File S1 Table A). This gene was also over-expressed in the amygdala of patients diagnosed with SSD (Chang et al., 2017).

Among the 16,175 genes tested for differential expression in the present study, the profile of several proinflammatory and neuroinflammatory genes were consistent with those from a candidate gene study of the amygdala from 9 days old mice exposed to MIA (Carlezon et al., 2019). Consistent with the patterns observed in mice exposed to viral mimetic Poly(I:C) MIA (Carlezon et al., 2019), an interaction between PRRSV-elicited MIA and sex was detected in pigs for the genes glial fibrillary acidic protein (GFAP), nitric oxide synthases 1 and 2 (NOS1 and NOS2, respectively), and translocator protein (TSPO)-associated protein (0.006 < *P* < 0.02). These patterns, albeit consistent, failed to surpass the FDR-adjusted *P* < 0.05 threshold. Also consistent with the MIA study of mice amygdala (Carlezon et al., 2019), the differences in expression between MPA and CON males for tumor necrosis factor alpha (TNF-α), and interleukin 1 beta (IL-1β), were not statistically significant. The expression levels for interleukin 6 (IL-6) and interleukin 1 beta (IL-1β) among MPA males were below the minimum threshold for testing, and therefore, the interaction effects for these genes are not reported. IL-6 and IL-1β were differentially expressed between MIA groups in female mice (Carlezon et al., 2019); however, the reported relative abundances for these genes suggests that MIA male mice, like MPA male pigs, presented the lowest levels of IL-6 and IL-1β abundance of all groups studied.

The pattern of several genes presenting a MIA-by-sex interaction effect was characterized by the same relative abundance between MIA groups, albeit sexes differed in magnitude (Table 1). Males presented a more extreme under-expression of CGA in MPA relative to CON than females (Table 1). This difference could be associated with the participation of CGA in multiple hormone processes that regulate female reproductive performance. The lower impact of MIA on the CGA levels in females may prevent the dysregulation of multiple downstream processes associated with reproductive function. Likewise, the under-expression of TTR in MPA relative to CON was more acute in males than in females (Table 1). Over-expression of TTR was noted in the amygdala of rats treated with MK-801, a N-methyl-D-aspartate antagonist that elicits SSD-like behavior (Matsuoka et al., 2008). The differential expressions of HBB and GBP1 among MIA groups detected in our study are also observed in the amygdala of SSD cases (Chang et al., 2017).

### Functional Analysis of Sex-Dependent Maternal Immune Activation Transcriptome

The study of over-represented functional categories among the genes presenting sex-dependent profiles between MPA and CON pigs (Tables 2, 3 and Supplementary File S1 Tables B, C) identified categories consistent with previous studies of MIA and amygdala inflammation. The enrichment of the KEGG pathways related to autoimmune disease and antigen processing and presentation via histocompatibility complex (MHC) (Table 2 and Supplementary File S1 Table B) are in agreement with the reported association between autoimmune diseases, SSD, and variants in the MHC gene family (Anders and Kinney, 2015). Autoimmune reactions are capable of inducing psychiatric symptoms that are mediated by the amygdala such as those associated with SSD (Lennox et al., 2012). Genes annotated to MHC receptor activity are over-expressed in the amygdala of individuals that have SSD (Chang et al., 2017). The cytokine-mediated signaling pathway was also over-represented among the genes under-expressed in MPA relative to CON pigs (Table 3). Consistent with our results, the expression of 28 genes annotated to immune stimulus had MIA-by-sex interaction effects in the microglia of GD 97 fetuses after GD 76 PRRSV injection (Antonson et al., 2019).

Multiple BPs associated with homeostasis and extracellular matrix assembly were enriched among the genes presenting a significant MIA-by-sex effect (Tables 2, 3 and Supplementary File S1 Tables B, C). This finding is in agreement with the deficit in perineuronal nets in the amygdala of patients diagnosed with SSD (Paylor et al., 2016). These nets are extracellular matrix structures that support the high metabolic demand of the interneurons, and contribute to ion homeostasis around them.

The enrichment of BP axon and brain development among the genes presenting an MIA-by-sex effect (Table 2 and Supplementary File S1 Table B) is consistent with a report that LPS-elicited MIA is associated with under-expression of neurodevelopmental genes in the rat fetal brain, including genes linked to ASD (Oskvig et al., 2012). Furthermore, the amygdala of rats prenatally exposed to valproic acid, a stressor that leads to ASD, presented activation of neuron development pathways (Barrett et al., 2017). Similarly, the KEGG pathway of cell-adhesion molecules (CAMs), molecules that are fundamental for nervous system development and maintenance, was enriched among the genes presenting a sex-dependent MIA effect. This pathway was also enriched among genes under-expressed in the brain of rats exposed to LPS-triggered MIA and among genes under-expressed in the cortex of patients diagnosed with ASD (Lombardo et al., 2018).

The KEGG pathway neuroactive ligand receptor interaction was enriched among the genes presenting a significant interaction effect characterized by under-expression in the amygdala of CON relative to MPA males (Table 3). This pattern is aligned with findings that Poly(I:C)-elicited MIA augmented the synaptic strength of glutamatergic projections from the frontal cortex into the amygdala of mice (Li et al., 2018). The neuroactive ligand receptor pathway encompasses neuroreceptor genes such as dopamine, serotonin, GABA, and glutamate receptors.

The impact of sex-dependent MIA effects on neuropeptide and hormone genes (e.g., CGA, POMC, and SSTR1) expressed in the amygdala is evidenced by the enrichment of GnRH signaling pathway and hormone activity among the genes under-expressed in MPA relative to CON pigs (Table 3 and Supplementary File S1 Table C). Our results suggest that the disruption of glucocorticoid hormone balance on the HPA axis initiated by MIA can have long-lasting effects because amygdala processes are regulated by glucocorticoid receptors and glucocorticoids repress GnRH secretion.

### Impact of Maternal Immune Activation on Gene Networks Within Sex

Further understanding of sex-dependent effects of MIA on the co-expression of gene sets was gained from the identification of WGCNA modules of genes that share relative expression profiles between the CON and MPA groups in males (Table 9) or that share relative expression profiles between sexes in MPA pigs (Table 10). The WGCNA gene modules profiling changes between MIA groups in males uncovered enrichment of genes annotated to AD (Table 9). This result is consistent with findings of common molecular mechanisms shared between SSD and AD (Prestia, 2011; Sumitomo et al., 2018). Likewise, the enrichment of ATP metabolic processes among the module of genes associated with MIA effects in males is in agreement with evidence that mitochondrial dysfunction associated with SSD (Prabakaran et al., 2004).

Insights into the distinct vulnerability to MIA between sexes on the interplay between critical genes was gained from the study of the network of genes that had a significant MIA-by-sex effect in the enriched neuroactive ligand receptor pathway. The comparison of Figures 1, 2 highlights the distinct interaction between genes in response to PRRSV in males and females, respectively. Notably, males present a strong and consistent over-expression (i.e., red color) of genes in CON relative to MPA, with the exception of SSTR1. On the other hand, females present a weaker expression differential with a slight majority of genes under-expressed (i.e., blue color) in CON relative to MPA pigs. These results suggest lower vulnerability to MIA effects on gene expression in females than in males at 3 weeks of age. An example of this pattern is the module of the neuropeptide receptors, angiotensin II receptor type 1 (AGTR1), and PTH1R. The co-expression of these two G-coupled receptors is consistent with the shared metabolic function (Regard et al., 2008). Distinct to the opposite patterns between sexes observed in the previous network cluster, the highly connected CGA and GH1 are over-expressed in CON relative to MPA in both sexes, albeit the differences are more extreme in males than in females. Correlated under-expression of GH1 and CGA was observed in the cerebellum and prefrontal cortex of rats that presented altered depressive-like behavior (Yamamoto et al., 2015).

### Sex-Independent Associations Between Maternal Immune Activation and Transcriptome Changes

The 161 differentially expressed genes between MPA and CON pigs (Table 4 and Supplementary File S1 Table D) included genes supported by previous studies of MIA. Additionally, the differential expression of several genes in Table 4 has been linked to neurological disorders such as SSD, ASD, and AD. The over-expression of ANKRD24 in MPA relative to CON pigs (Table 4) is supported by the over-expression of an ankyrin repeat domain family member (ANKRD32) in rat fetal brains exposed to MIA elicited by LPS (Oskvig et al., 2012). IFITM3, IFITM1, and CTSC were over-expressed in the amygdala of MPA relative to CON pigs (Table 4). Similarly, these genes were over-expressed in the amygdala of individuals diagnosed with SSD (Takao et al., 2012; Chang et al., 2017). Consistent with our results, IFITM3 was over-expressed in the hippocampi of neonatal mice treated with Poly(I:C) that resulted in developmental impairment of the central nervous system and lasting brain dysfunction. Conversely, *Ifitm3^–/–^* mice treated with Poly(I:C) exhibited normal neural development and did not present neural deficiencies (Ibi et al., 2013). CTSC was also over-expressed in the hippocampus of Shn-2 KO mice that exhibited SSD-like behaviors (Takao et al., 2012). MAP2K7 was over-expressed in MPA relative to CON pigs (Table 4), and this gene has been implicated in SSD incidence. MAP2K7 exclusively activates c-Jun N-terminal kinases (JNK) (Lisnock et al., 2000; Zeke et al., 2016), a mediator of the MIA response in the developing fetus that likely contributes to the neurological abnormalities in SSD (Openshaw et al., 2019).

HS3ST5 and SPP1 were over-expressed in the amygdala of MPA compared to CON pigs (Table 4), and both genes have been linked to ASD. Consistent with the patterns in our study, the expression of SPP1 in the temporal cortex of humans was higher in individuals diagnosed with ASD compared to controls (Garbett et al., 2008). Indeed, SPP1 participates in multiple immuno-related pathways in neural tissues (Carecchio and Comi, 2011; Brown, 2012). Genome-wide association studies identified a genetic variation near HS3ST5 that was significantly associated with ASD while a single-nucleotide polymorphism within this gene has been associated with SSD (Wang et al., 2009, 2015).

TACC1, CRABP2, and ALDH1A1 participate in the retinoid signaling and metabolic pathways and were differentially expressed in MPA compared to CON pigs (Table 4 and Supplementary File S1 Table D). Dysregulation of retinoid pathways may disrupt neural development leading to SSD (Goodman, 1996), and retinoid toxicity and deficiency are associated with central nervous system abnormalities (Maden, 1994). The differential expression of genes in the retinoid pathways is consistent with the previously described changes in the expression of genes in the thyroid hormone cascades. Defective cross talks between the retinoid and/or thyroid hormone processes have been associated with the development of SSD (Palha and Goodman, 2006). CRABP2 was under-expressed in MPA compared to CON pigs (Supplementary File S1 Table D), and mutations in this gene have been implicated in SSD (Goodman, 1995). Aldehyde dehydrogenase 1 family member A1 (ALDH1A1) was under-expressed in MPA compared to CON pigs (Table 4). ALDH1A1 was under-expressed in the amygdala of neonatal rats exposed to odor-shock conditioning, mimicking the effects of unpredictable early life trauma and resulting in amygdala dysfunction (Sarro et al., 2014).

IGF2 was under-expressed in MPA compared to CON pigs (Table 4), and consistent with our results, the systemic administration of IGF2 reduces ASD phenotypes; promotes normal social interaction, cognition, and executive function; and reduces repetitive behavior (Steinmetz et al., 2018). TTR was over-expressed in the amygdala of MPA compared to CON pigs. Over-expression of TTR was noted in the amygdala of rats treated with MK-801, a N-methyl-D-aspartate antagonist that elicits SSD-like behavior (Matsuoka et al., 2008).

### Functional Analysis of Sex-Independent Maternal Immune Activation Transcriptome

The functional categories over-represented among the genes differentially expressed between MPA and CON pigs (Table 5 and Supplementary File S1 Tables E) were supported by previous studies of MIA and associated neurodevelopmental disorders. Among these categories was the BP anatomical structure formation involved in morphogenesis and angiogenesis (Table 5). This result is consistent with the under-expression of early growth response 1 (EGR1, a regulator of angiogenic factors) in the GD 97 fetal microglia of MPA relative to CON pigs of both sexes after the GD 76 PRRSV challenge (Antonson et al., 2019). Angiogenesis was also enriched among differentially expressed genes in the frontal cortex of rats that are exposed to valproic acid *in utero* (Hill et al., 2015) to model ASD (Favre et al., 2013). The observed enrichment of the BP angiogenesis was also reported among genes dysregulated in the hippocampal microglia of mice exposed to Poly(I:C) relative to control mice (Mattei et al., 2017).

Several KEGG pathways associated with inflammation and infection, including allograft rejection, Epstein–Barr virus infection, and autoimmune thyroid disease, were enriched, among the genes differentially expressed between MPA and CON pigs (Table 5 and Supplementary File S1 Table E). This finding is consistent with the significant effect of MIA from GD 76 PRRSV injection on expression of 12 genes in the amygdala of GD 97 fetuses from both sexes (Antonson et al., 2019). At this fetal stage, most of the genes differentially expressed between MPA and CON gilts were annotated to the BP immune response, including genes annotated to the BP cytokine-mediated signaling pathway, and to the toll-like receptor pathway (Antonson et al., 2019). This enrichment is in agreement with reports of amygdala inflammation and transcriptome changes in glial cells in response to MIA elicited by LPS administration in mice that persist into adulthood (O’Loughlin et al., 2017).

Many BPs that modulate neurodevelopment were enriched among genes under-expressed in MPA compared to CON pigs, including microtubule bundle formation, cilium morphogenesis, cilium organization, cilium movement, and axoneme assembly (Table 6 and Supplementary File S1 Table F). Reduced neuronal primary cilia can reduce cellular communication during development, the number of dendrites (Goetz and Anderson, 2010; Guadiana et al., 2013), and can hinder neurogenesis in the adult brain (Amador-Arjona et al., 2011). Furthermore, primary cilia participate in the development of the circuitry of GABAergic interneurons, and disrupted cilia formation leads to dysregulated excitatory/inhibitory signaling between neurons (Guo et al., 2017). This finding is consistent with the glutamatergic and GABAergic-associated genes that presented a significant MIA-by-sex interaction effect previously discussed (Table 1). Disruption of this circuitry underlies the neurological disorders associated with ASD and SSD (Bourgeron, 2009; Marin, 2012; Li et al., 2016), and olfactory neuronal precursors collected from SSD patients were found to have less primary cilia growth *in vitro* compared to controls (Munoz-Estrada et al., 2018).

### Main Effect of Sex on Gene Expression and Functional Enrichment

The amygdala is a sexually dimorphic area of the brain that is highly responsive to signaling from gonadal steroid hormones (Hines et al., 1992; Cooke and Woolley, 2005). Supporting this, 150 genes were differentially expressed (Table 7 and Supplementary File S1 Table G), and most were over-expressed in males compared to females. The activity of the amygdala has been related to cortisol response, immune responses, and hormonal physiology that differs between sexes (Goldstein et al., 2019). The amygdala is a brain region dense in sex steroid, glucocorticoid, and cytokine receptors that are key co-activators of the HPA axis. The amygdala of male mice has more excitatory synapses per neuron compared to female mice (Cooke and Woolley, 2005; Guneykaya et al., 2018).

The enriched KEGG pathway neuroactive ligand–receptor interaction and biological process response to hormone (Table 8 and Supplementary File S1 Table H) are supported by the evidence indicating that the amygdala development is greatly influenced by sex hormones (Cooke and Woolley, 2005). LHX9 and LHB were strongly over-expressed, while IGSF1 was slightly over-expressed in males compared to females (Table 7). The importance of LHX9 in the sexual dimorphism of males and females is highlighted by evidence that *Lhx9^–/–^* mice fail to produce gonads, and male *Lhx9^–/–^* resemble females phenotypically (Birk et al., 2000). IGSF1-deficient males have later testosterone secretion than individuals with normal IGSF1 expression (Joustra et al., 2013).

Several developmental BP including gland, embryo, sensory organ, and brain development, along with the MF extracellular matrix structural constituent, were enriched among the genes differentially expressed between sexes (Table 8). Supporting these categories, EIF1AY was over-expressed in the amygdala of males compared to females (Table 7). Consistent with our results, EIF1AY was over-expressed in the male brain at early childhood, puberty, and adulthood and contributes to the structural sexual dimorphism of the amygdala (Shi et al., 2016).

### Additional Considerations

The results of our study of long-lasting changes in the amygdala transcriptome profiles at approximately 60 days after exposure to PRRSV advanced the understanding of the impact of MIA on molecular pathways associated with neurodevelopmental processes, neurodegenerative diseases, and behavior disorders. Biological processes impacted at in the amygdala of PD 22 pigs were also impacted at other developmental stages such as fetal GD 83, 96, or 111 (Antonson et al., 2018, 2019) and included antigen processing and presentation of peptide or polysaccharide antigen via MHC class II and immune response. Likewise, neurodevelopmental processes impacted in the present study were also reported in the amygdala of rats prenatally exposed to MIA by means of valproic acid (Barrett et al., 2017).

Notably, we identified sex-dependent vulnerability to the effects of MIA on gene expression in the amygdala of pigs at PD 22. Sex-independent effects dominated in GD 96 fetuses (Antonson et al., 2019). This comparison supports the hypothesis that sex-dependent effects of MIA in the amygdala become stronger as the animal develops. Additional understanding of MIA effects on the amygdala requires the evaluation of female and male pigs at older ages, in consideration that female pigs reach puberty at 5 months of age. Also, evaluation of the impact of MIA on other brain structures that interact with the amygdala will enable the determination of broader molecular profiles.

## Conclusion

The present study advances the understanding of the prolonged effects of MIA in the molecular pathways of the amygdala, a brain structure key to social, feeding, and other behaviors. The RNA-Seq profiling of 3-week-old female and male pigs, 2 months after viral infection during gestation, offered insights into MIA-associated neurodevelopmental diseases in humans such as ASD and SSD and potential effects in livestock health. The prevalent and sex-dependent dysregulation of genes in immune pathways was detected, supporting established immunotherapies to alleviate the pathophysiology of SSD, ASD, and AD (Busche et al., 2015; Miller and Buckley, 2016).

Our study detected lesser explored molecular processes affected by MIA including the neuroactive ligand–receptor, glutamatergic, amyloid peptides, neuropeptide, retinoid, and ciliogenesis systems. The detection of the previous processes backs the integration of therapies based on immune modulation together with therapies that target neurochemical dysfunction in MIA-associated disorders. The effectiveness of these therapies may be further advanced by our innovative identification of frequently disrupted neuropeptide systems. Our functional and network analyses solidify the promise of multifactorial therapeutic strategies combining immune and neurochemical targets to ameliorate MIA-associated neurodevelopmental and neurodegenerative disorders.

## Data Availability Statement

The datasets presented in this study can be found in online repositories. The names of the repository/repositories and accession number(s) can be found below: https://www.ncbi.nlm.nih.gov/, GSE149695.

## Ethics Statement

The animal studies were approved by the Illinois Institutional Animal Care and Use Committee (IACUC) at the University of Illinois, and are in compliance with the USDA Animal Welfare Act and the NIH Public Health Service Policy on the Humane Care and Use of Animals.

## Author Contributions

RJ and SR-Z contributed to the conception and design of the study. CB, AA, LR, HR, MC, AKH, and AGH organized the animal experiments and collected the data. MK, PZ, and BS performed the bioinformatics analyses. MK and SR-Z interpreted the results and wrote the first draft of the manuscript. All authors contributed to manuscript revision, read, and approved the submitted version.

## Conflict of Interest

The authors declare that the research was conducted in the absence of any commercial or financial relationships that could be construed as a potential conflict of interest.
